# Studies on lipid A isolated from *Phyllobacterium trifolii* PETP02^T^ lipopolysaccharide

**DOI:** 10.1007/s10482-017-0872-0

**Published:** 2017-04-13

**Authors:** Katarzyna Zamlynska, Iwona Komaniecka, Kamil Zebracki, Andrzej Mazur, Anna Sroka-Bartnicka, Adam Choma

**Affiliations:** 1 Department of Genetics and Microbiology, Maria Curie-Sklodowska University, Akademicka 19, 20-033 Lublin, Poland; 2 Department of General Microbiology, Maria Curie-Sklodowska University, Akademicka 19, 20-033 Lublin, Poland

**Keywords:** 2,3-Diamino-2,3-dideoxy-d-glucose, Lactobacillic acid, Lipid A structure, Lipopolysaccharide, *Phyllobacterium trifolii*

## Abstract

The structure of lipid A from lipopolysaccharide of *Phyllobacterium trifolii* PETP02^T^, a nitrogen-fixing symbiotic bacterium, was studied. It was found that the lipid A backbone was composed of two 2,3-diamino-2,3-dideoxy-d-glucose (Glc*p*N3N) residues connected by a β-(1 → 6) glycosidic linkage, substituted by galacturonic acid (Gal*p*A) at position C-1 and partly decorated by a phosphate residue at C-4′ of the non-reducing Glc*p*N3N. Both diaminosugars were symmetrically substituted by 3-hydroxy fatty acids (14:0(3-OH) and 16:0(3-OH)). Ester-linked secondary acyl residues [i.e. 19:0cyc and 28:0(27-OH) or 28:0(27-4:0(3-OMe))] were located in the distal part of lipid A. A high similarity between the lipid A of *P. trifolii* and *Mesorhizobium* was observed and discussed from the perspective of the genetic context of both genomes.

## Introduction

The genus *Phyllobacterium* was originally described by Knösel (1962), mainly on the basis of phenotypic features of bacteria developing within leaf nodules of tropical ornamental plants. The genus comprised two species, *Phyllobacterium myrsinacearum* and *Phyllobacterium rubiacearum* (Knösel 1984), which were merged under the emended description (Mergaert et al. 2002). Currently, the genus *Phyllobacterium* belongs to the family *Phyllobacteriaceae* in the order *Rhizobiales* of the class *alpha*-*Proteobacteria* and contains ten species: *P. myrsinacearum* (Mergaert et al. 2002), *P. trifolii* (Valverde et al. 2005), *P. catacumbae* (Jurado et al. 2005), *P. bourgognense*, *P. ifriqiyense*, *P. leguminum, P. brassicacearum* (Mantelin et al. 2006), *P. endophyticum* (Flores-Fèlix et al. 2013), *P. loti* (Sánchez et al. 2014), and *P. sophorae* (Jiao et al. 2015).

*Phyllobacterium trifolii* PETP02^T^ is the type strain of the species. It has been isolated from nodules of *Trifolium pratense* belonging to natural clover plants of north-west Spain. A comparison of the 16S rRNA gene sequence of *P. trifolii* indicated that it is closely related to the members of the genus *Mesorhizobium*. These bacteria can establish an effective symbiosis with *Trifolium* and *Lupinus,* plants that differ with regard to the nodule morphology. This property is rare among symbiotic bacteria. The *P. trifolii nodD* gene sequence shows high similarity (99%) to the homologous genes of *Rhizobium etli* CFN32^T^ and *Ochrobactrum* sp. LUP21, bacteria inducing a determinate type of root nodules on respective legumes, whereas nodules observed on *Trifolium,* the major macrosymbiont of *P. trifolii,* represent the indeterminate type (Valverde et al. 2005; Trujillo et al. 2005). The signal molecules (Nod factors) of *Rhizobium leguminosarum* bv. Trifolii and *Bradyrhizobium,* typical microsymbionts of clover and lupine, respectively, possess different fine structures. This evidence suggests that *Trifolium* and *Lupinus* do not select their microsymbionts strictly and that the host plant receptors could be unspecific (D’Haeze and Holsters 2002; Soulemanov et al. 2002; Schlaman et al. 2006; Renier et al. 2011). Like most Gram-negative bacteria, *P. trifolii* synthesizes lipopolysaccharide (LPS). The LPS molecules occupy at least 75% of the bacterial cell surface and are composed of three distinct domains: lipid A, core oligosaccharide, and O-specific polysaccharide (O-PS). The domains differ in terms of their structure and biosynthesis pathways (Silipo et al. 2010). Lipid A is a main constituent of the outer leaflet of the outer membrane (OM) and anchors entire LPS in OM through electrostatic and hydrophobic interactions, while the carbohydrate domains of LPS are oriented outwards (Raetz and Whitfield 2002). *P. trifolii* PETP02^T^ synthesizes mainly the smooth (S) form of LPS, and its O-PS structure has been described (Zamlynska et al. 2015). The strain synthesized two types of O-polysaccharides, containing hexa- and disaccharide repeats, respectively. The proper structure of the entire LPS determines appropriate integrity and flexibility of the outer membrane, essential for the correct morphology and functionality of bacteroids—the endosymbiotic form of rhizobia in which nitrogen fixation takes place. LPS delays or completely blocks the hypersensitivity reaction (HR) induced by rhizobia and suppresses systemic acquired resistance (SAR) during bacterial infection of root nodules (Dow et al. 2000; Albus et al. 2001; Menezes and Jared 2002; Mathis et al. 2005). For a long time, it was believed that lipid A was not important for development of an effective symbiosis. However, the data published during recent years have shown that mutants in genes encoding lipid A biosynthesis were more sensitive to pH and osmolarity changes, grew slowly, and revealed delayed nodule development. Also, the symbiotic effectiveness of these mutants, measured by nitrogen fixation abilities, was considerably reduced (Vedam et al. 2004; Ferguson et al. 2005; Hagg et al. 2009; Choma et al. 2017).

The structure of rhizobial lipids A differ considerably from the model one of *Escherichia coli*. The enterobacterial glucosamine-based hexa-acylated lipid A has endotoxic activity and is the most potent agonist of innate immunity in humans (Zähringer et al. 1994; Beutler and Rietschel 2003). Respective lipids A from *Rhizobium* and *Sinorhizobium* genera contain backbones based on glucosamine and 2-aminogluconate (Bhat et al. 1994; Que et al. 2000a, b; Kannenberg and Carlson 2001; Jeyaretnam et al. 2002; Gudlavalleti and Forsberg 2003; Ferguson et al. 2005, 2006), whereas the disaccharide backbone in other nodule-forming bacteria is composed exclusively of 2,3-diamino-2,3-dideoxy-d-glucose (Glc*p*N3N) (Choma and Sowiński 2004; Komaniecka et al. 2010; Choma et al. 2012; Brown et al. 2013; Komaniecka et al. 2014; Silipo et al. 2014). Sugar backbones of rhizobial lipids A are substituted by amide- and ester-linked 3-hydroxy fatty residues, whose hydroxyl groups can be acylated by other non-polar and (ω-1)-hydroxy/oxo very long chain fatty acid (VLCFA). For example, 27-hydroxyoctacosanoic acid is most often found in lipids A of members of alpha-rhizobia (except for the *Azorhizobium* genus) and is considered as chemotaxonomic marker of this group of bacteria (Bhat et al. 1991a, b). These very long chain fatty acids could be partially acylated by 3-hydroxybutyric or 3-methoxybutyric acids or by one or two hopanoid residues in some bradyrhizobial lipids A (Komaniecka et al. 2014; Silipo et al. 2014; Choma et al. 2017).

The structures of lipids A isolated from symbiotic bacteria classified outside of the *Rhizobiaceae* and *Bradyrhizobiaceae* families, such as that of the *Phyllobacterium* genus, have been studied sporadically, up to now. The aim of this article was to fill the gap and describe the structure of lipid A from *P. trifolii* PETP02^T^ in detail.

## Materials and methods

### Bacterial culture conditions and lipopolysaccharide isolation

The *P. trifolii* PETP02^T^ strain was obtained as a kind gift from Dr. E. Velazquez, from the University of Salamanca, Spain. The bacteria were cultivated for 7 days in a liquid Tryptone Yeast medium at 28 °C with aeration by vigorous shaking. The bacteria were harvested and the cell pellet was washed twice with saline. Bacterial mass was subjected to delipidation and enzymatic digestion procedures according to the method described in detail by Choma et al. (2012). The lipopolysaccharide was extracted from degraded cells using a hot 45% phenol/water method (Westphal and Jann 1965; Johnson and Perry 1976) and purified by ultracentrifugation, as described by Zamlynska et al. (2015).

### Lipid A isolation and *O*-deacylation

Lipid A was cleaved from LPS by mild acid hydrolysis (aq 1% acetic acid, 100 °C, 3 h). After cooling, the hydrolysate was converted to the two-phase Bligh-Dyer system and the chloroform phase containing the lipid A portion was separated by centrifugation (4000 × g, 15 min, 20 °C), collected, and washed with the water phase from a freshly prepared two-phase Bligh–Dyer mixture (Que et al. 2000a; Choma et al. 2012). The lipid A preparation obtained was stored at −20 °C in chloroform/methanol (2:1, v/v). Lipid A was *O*-deacylated by incubation of a 1 mg sample in chloroform/methanol/1 M aqueous NaOH, 2:3:1 (v/v/v), for 1 h, at room temperature, according to the method described previously (Que-Gewirth et al. 2004; Komaniecka et al. 2014). Partial lipid A *O*-deacylation was performed using 25% ammonium hydroxide (Lukasiewicz et al. 2010).

### Chemical analyses of lipid A

The sugar composition was established by hydrolysis of lipid A with 4 M HCl (100 °C, 4 h) and conversion of liberated monosaccharides into (amino)alditol acetates (Sawardeker et al. 1965). The absolute configuration of the monosaccharides was established by analysis of trimethylsilylated R-(-)-butyl glycosides according to a modified procedure developed by Gerwig et al. (1978). The fatty acid composition was established after methanolysis of lipid A (2 M HCl/MeOH, 85 °C, 18 h) and conversion of the obtained hydroxy fatty acid methyl esters into trimethylsilyl derivatives. 3-Hydroxy fatty acids were converted to l-phenylethylamides of 3-methoxy derivatives as described previously (Rietschel et al. 1976, Wollenweber et al. 1980) for analysis of the absolute configuration. Sugars and fatty acid derivatives were analysed using a gas chromatograph (Agilent Technologies, instrument 7890A) connected to a mass selective detector (Agilent Technologies MSD 5975C, inert XL EI/CI) (GLC-MS), using helium as a carrier gas. The chromatograph was equipped with a HP-5MS column (30 m × 0.25 mm). The temperature program was as follows: 150 °C for 5 min raised to 310 °C (5 °C min^−1^), and the final temperature was maintained for 10 min. l-Phenylethylamides of 3-methoxy fatty acid derivatives were also analysed isothermally (270 °C) using the same column. Heptadecanoic acid and 3-hydroxy tetradecanoic acids were used as standards for quantitative determination of fatty acids.

### NMR spectroscopy

The NMR spectra were recorded at 30 °C using a Varian Inova 500 instrument and a standard Varian software. The sample was dissolved in a mixture of CDCl_3_/CD_3_OD (1:1, v/v) with a drop (5 µl) of D_2_O. 1D (^1^H and ^31^P NMR) and 2D NMR spectra (^1^H,^1^H COSY, DQF-COSY, TOCSY, NOESY, ROESY) were recorded. Proton chemical shifts were measured in relation to TMS as an internal standard (δ_H_ 0.00). Phosphorous chemical shifts were measured relative to an external standard of 85% (v/v) phosphoric acid at δ_P_ 0.00 p.p.m.

### MALDI-TOF MS and MS/MS spectrometry

Matrix-assisted laser desorption/ionization time-of-flight mass spectrometry (MALDI-TOF–MS) was performed using a Waters SYNAPT G2-S*i* HDMS instrument (Waters Corporation, Milford, MA, USA) equipped with a 1 kHz Nd:YAG laser system. Acquisition of the data was performed using MassLynx software version 4.1 SCN916 (Waters Corporation, Wilmslow, United Kingdom). Spectra were recorded in positive and negative ion polarities. For MS/MS experiments, isolated precursor ions were fragmented using collision voltage of 60 V. Data were collected for 120 s for each ion separately. Mass spectra were assigned with a multi-point external calibration using red phosphorous (Sigma).

The lipid A sample was dissolved in chloroform/methanol (2:1, v/v) at a concentration of 10 µg/µl and one microliter of the sample was transferred into the target plate wells covered with a thin matrix film. The matrix solution was prepared from 2′,4′,6′-trihydroxyacetophenone (THAP) (200 mg/ml in methanol) mixed with nitrocellulose (NC) (15 mg/ml, suspended in 2-propanol/acetone (1:1, v/v)) in proportion of 4:1 (v/v), as previously described by Silipo et al. (2005).

### Bioinformatics tools

Standard BLASTP (with the cut-off *E*-value of 10^−5^) was used in searching for putative proteins engaged in the biosynthetic pathway of *Phyllobacterium* lipid A. *Mesorhizobium loti* MAFF 303099 protein sequences were used as queries in BLASTP searches against five *Phyllobacterium* strains registered in the Genomes OnLine Database. Individual protein sequences were then compared across their entire span with an on-line Global Alignment tool (using the Needleman-Wunsch algorithm) provided by the National Center for Biotechnology Information (NCBI).

## Results

### Structural analysis of the lipid A preparation from *P. trifolii*

Delipidated *P. trifolii* cells were extracted with 45% hot phenol in water and LPS was found mainly in the water phase. The lipid A fraction was obtained by mild hydrolysis of LPS using 1% acetic acid in water. Galacturonic acid (Gal*p*A) and Glc*p*N3N were identified as the only sugar components of lipid A. Their absolute configuration was shown to be d. Fatty acid analysis revealed the presence of 14:0(3-OH), 15:0(3-OH), 16:0(3-OH), 17:0(3-OH), and 18:0(3-OH) acids (Table 1). All of them were amide-linked and had the d absolute configuration. d-3-Hydroxy acids are characteristic components of all lipopolysaccharides described so far (Rietschel 1976). 3-Hydroxytetradecanoic and 3-hydroxyhexadecanoic acids were the main 3-hydroxy fatty acids in lipid A. Two long chain (ω-1)-hydroxy fatty acids (28:0(27-OH) and 30:0(29-OH)) as well as their oxo-analogues (28:0(27-oxo) and 30:0(29-oxo)) were present as ester-linked substituents of lipid A. Among ester-linked acids, non-polar fatty acids (16:0, 18:0, 18:1, and 19:0cyc) were found. The lactobacillic acid (19:0cyc) was the most abundant. This component was identified based on its chromatographic properties (retention time), compared with an authentic lactobacillic methyl ester. Cyclopropane ring-containing fatty acids give characteristic series of artificial components after methanolysis (Orgambide et al. 1993). All these artificial fatty acids were found in the solvolysed (in methanolic HCl) lipid A preparation (data not shown).

**Table 1:** Fatty acid components of *P. trifolii* PETP02^T^ LPS

Component	Amount [µg/mg]
**14:0**(**3-OH**)	**20.8**
15:0(3-OH)	3.7
**16:0**(**3-OH**)	**13.7**
17:0(3-OH)	1.6
18:0(3-OH)	1.2
16:0	4.2
18:0	2.3
18:1	5.4
**19:0cyc**	**28.3**
**28:0**(**27-OH**)	**25.7**
28:0(27-oxo)	2.1
30:0(29-OH)	2.2
30:0(29-oxo)	2.3

The bolded fatty acids are the most abundant

**Fig. 1:**
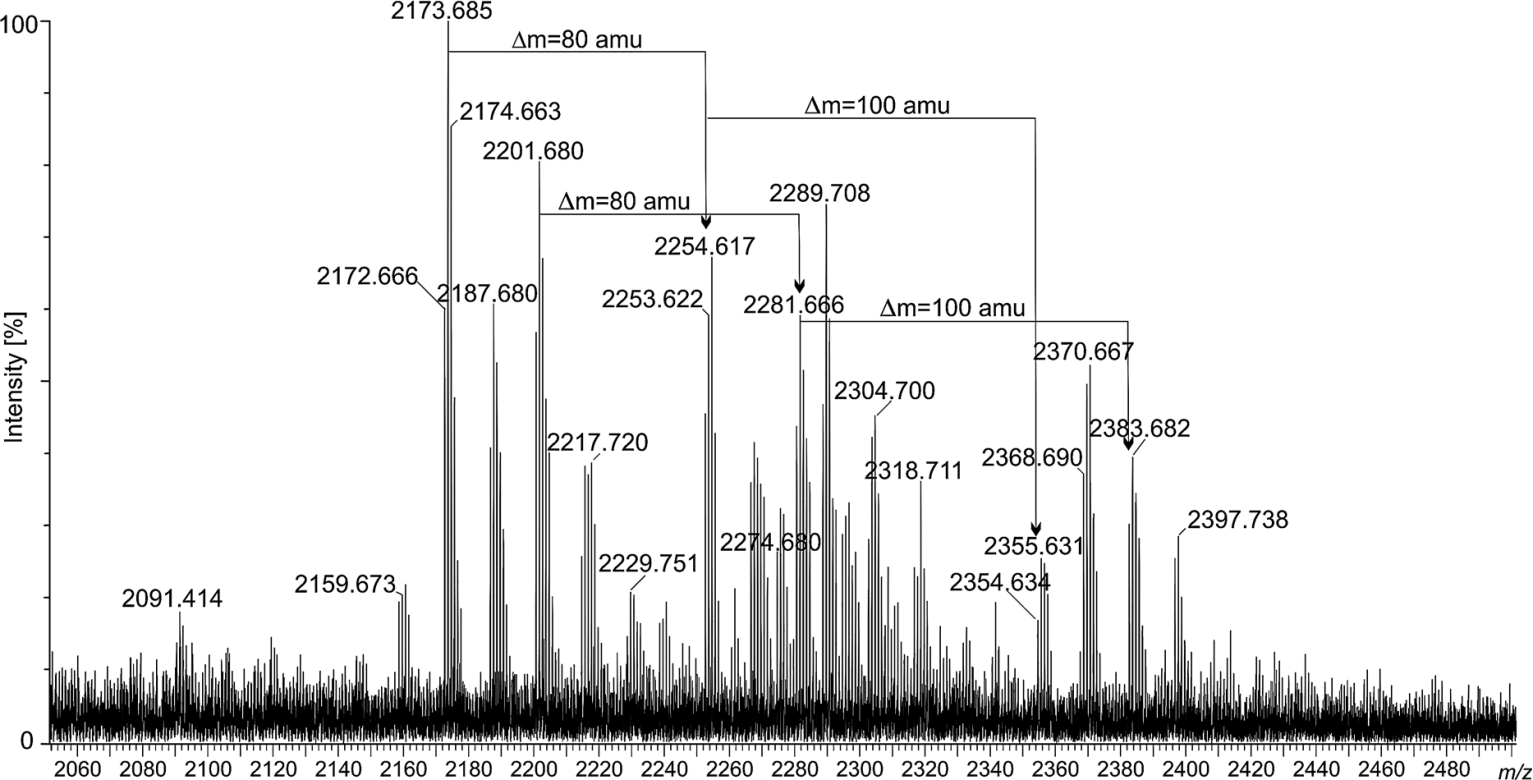
Negative ion mode of MALDI-TOF–MS spectrum obtained for the intact lipid A sample of *P. trifolii* PETP02^T^. The mass differences of 80 amu and 100 amu between lipid A species refer to the presence/absence of phosphate and 3-*O*-methoxybutyryl residues, respectively

**Fig. 2:**
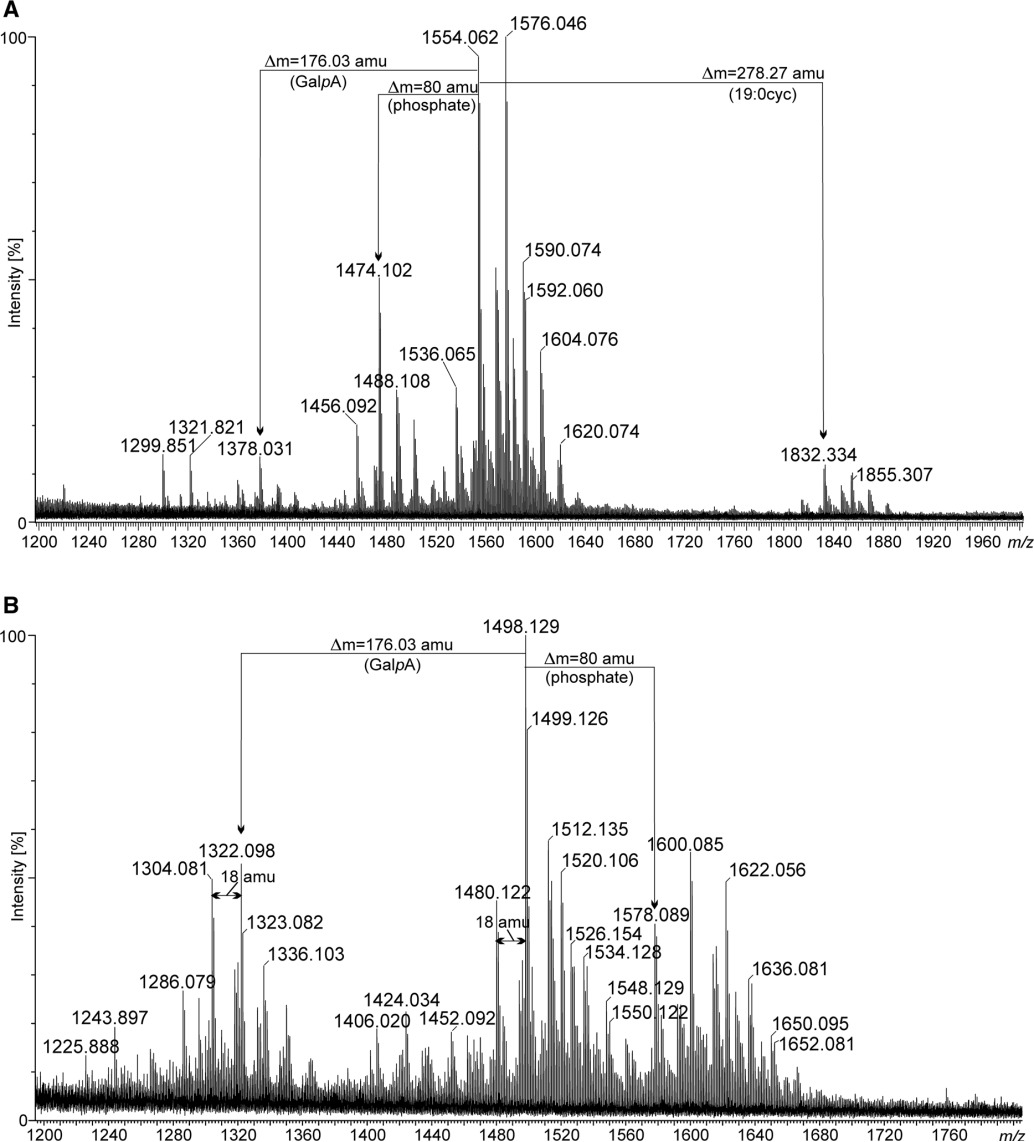
MALDI TOF MS spectra in negative (**a**) and positive (**b**) ion modes of *P. trifolii* PETP02^T^ lipid A *O*-deacylated with 1 M NaOH

An aliquot of lipid A was analysed by negative ion mode MALDI-TOF spectrometry (Fig. 1). Lipid A showed significant heterogeneity due to the 3-OH-fatty acid variability (Table 2). The heaviest group of ions was found between *m/z* at 2354.6 and 2405.0 showing the presence of a hexaacylated lipid A species composed of two Glc*p*N3N residues, uronic acid, phosphate residue, 28:0(27-(4:0(3-OCH_3_)) acyloxyacyl residue, 19:0cyc, and 3-hydroxy-acyl chains with different length (Δ mass = 14 amu). Ions representing lipid A species devoid of phosphate (Δ mass = 80 amu) and 3-*O*-methoxybutyryl (Δ mass = 100 amu) were placed on the other side of the spectrum (signals between *m/z* at 2159.6 and 2229.7). The middle part of the spectrum is the most crowded region. This group of ions comprises lipid A species modified with phosphate but not with 3-*O*-methoxybutyryl and vice versa (3-*O*-methoxybutyryl-containing, but deprived of phosphate). A number of lipid A molecules bearing a 28:0(27-oxo) residue were found in this region. They were always decorated with phosphate. Negative and positive ions derived from the *O*-deacylated lipid A are depicted in Fig. 2a, b. Negative ions representing *O*-deacylated lipid A molecules are in the *m/z* range from 1225.8 to 1665.0 (Fig. 2a, b). The less abundant group of ions around *m/z* at 1855 in the negative ion mode spectrum can be treated as not fully *O*-deacylated lipid A (Fig. 2a). The mass difference of 278.2 amu pointed to the lactobacillic residue attached to the primary 3-hydroxy fatty acid in lipid A. Three groups of ions (around *m/z* at 1600, 1498, and 1322) can be classified to (i) complete, (ii) dephosphorylated, and (iii) deprived of both phosphate and galacturonic acid residue *O*-deacylated lipid A, respectively (Fig. 2a, b and Table 2). The difference in the mass of the intact and *O*-deacylated lipid A molecules confirmed the previously mentioned findings that both 28:0(27-OH/oxo) and 19:0cyc constitute ester linked secondary residues.

**Table 2:** Masses and proposed compositions of selected ions observed in MALDI-TOF MS of intact and *O*-deacylated lipid A isolated from *P. trifolii* PETP02^T^

No.	Observed ion (*m/z*)	Type of ion	Calculated *m/z* values	Acyl substitution pattern	Proposed composition	Comments
1.	2172.666	[M−H]^−^	2172.6984	Hexaacyl	2 × GlcN3N 1 × GalA 2 × 16:0(3-OH) 2 × 14:0(3-OH) 1 × 28:0(27-oxo) 1 × 19:0cyc	Intact lipid A Negative ion mode
2.	2174.663	[M−H]^−^	2174.7140	Hexaacyl	2 × GlcN3N 1 × GalA 2 × 16:0(3-OH) 2 × 14:0(3-OH) 1 × 28:0(27-OH) 1 × 19:0cyc	Intact lipid A Negative ion mode
3.	2254.617	[M−H]^−^	2254.6804	Hexaacyl	2 × GlcN3N 1 × GalA 2 × 16:0(3-OH) 2 × 14:0(3-OH) 1 × 28:0(27-OH) 1 × 19:0cyc 1 × P	Intact lipid A Negative ion mode
4.	2274.680	[M−H]^−^	2274.7665	Hexaacyl	2 × GlcN3N 1 × GalA 2 × 16:0(3-OH) 2 × 14:0(3-OH) 1 × 28:0(27-oxo) 1 × 19:0cyc 1 × 4:0(3-OCH_3_)	Intact lipid A Negative ion mode
5.	2354.634	[M−H]^−^	2354.7327	Hexaacyl	2 × GlcN3N 1 × GalA 2 × 16:0(3-OH) 2 × 14:0(3-OH) 1 × 28:0(27-oxo) 1 × 19:0cyc 1 × 4:0(3-O CH_3_) 1 × P	Intact lipid A Negative ion mode
6.	2196.649	[M+Na]^+^	2196.6949	Hexaacyl	2 × GlcN3N 1 × GalA 2 × 16:0(3-OH) 2 × 14:0(3-OH) 1 × 28:0(27-oxo) 1 × 19:0cyc 1 × Na	Intact lipid A Positive ion mode
7.	2198.675	[M+Na]^+^	2198.7105	Hexaacyl	2 × GlcN3N 1 × GalA 2 × 16:0(3-OH) 2 × 14:0(3-OH) 1 × 28:0(27-OH) 1 × 19:0cyc 1 × Na	Intact lipid A Positive ion mode
8.	2226.681	[M+Na]^+^	2226.7418	Hexaacyl	2 × GlcN3N 1 × GalA 3 × 16:0(3-OH) 1 × 14:0(3-OH) 1 × 28:0(27-oxo) 1 × 19:0cyc 1 × Na	Intact lipid A Positive ion mode
9.	2278.597	[M+Na]^+^	2278.6769	Hexaacyl	2 × GlcN3N 1 × GalA 2 × 16:0(3-OH) 2 × 14:0(3-OH) 1 × 28:0(27-OH) 1 × 19:0cyc 1 × Na 1 × P	Intact lipid A Positive ion mode
10.	2328.689	[M+2Na−H]^+^	2328.6901	Hexaacyl	2 × GlcN3N 1 × GalA 3 × 16:0(3-OH) 1 × 14:0(3-OH) 1 × 28:0(27-oxo) 1 × 19:0cyc 2 × Na 1 × P	Intact lipid A Positive ion mode
11.	1378.031	[M−GalA−H]^−^	1377.9751	Tetraacyl	2 × GlcN3N 2 × 16:0(3-OH) 2 × 14:0(3-OH)	*O*-deacylated lipid A Negative ion mode
12.	1474.102	[M−H]^−^	1474.0407	Tetraacyl	2 × GlcN3N 1 × GalA 2 × 16:0(3-OH) 2 × 14:0(3-OH)	*O*-deacylated lipid A Negative ion mode
13.	1554.062	[M−H]^−^	1554.0070	Tetraacyl	2 × GlcN3N 1 × GalA 2 × 16:0(3-OH) 2 × 14:0(3-OH 1 × P	*O*-deacylated lipid A Negative ion mode
14.	1576.046	[M+Na−2H]^−^	1575.9889	Tetraacyl	2 × GlcN3N 2 × 16:0(3-OH) 2 × 14:0(3-OH) 1 × P 1 × Na	*O*-deacylated lipid A Negative ion mode
15.	1832.334	[M−H]^−^	1832.2680	Pentaacyl (incomplete deacylation)	2 × GlcN3N 1 × GalA 3 × 16:0(3-OH) 1 × 14:0(3-OH) 1 × 19:0cyc 1 × P	*O*-deacylated lipid A Negative ion mode
16.	1322.098	[M−GalA+Na]^+^	1322.0051	Tetraacyl	2 × GlcN3N 2 × 16:0(3-OH) 2 × 14:0(3-OH) 1 × Na	*O*-deacylated lipid A Positive ion mode
17.	1498.129	[M+Na]^+^	1498.0372	Tetraacyl	2 × GlcN3N 1 × GalA 2 × 16:0(3-OH) 2 × 14:0(3-OH) 1 × Na	*O*-deacylated lipid A Positive ion mode
18.	1512.135	[M+Na]^+^	1512.0528	Tetraacyl	2 × GlcN3N 1 × GalA 2 × 16:0(3-OH) 1 × 14:0(3-OH) 1 × 15:0(3-OH) 1 × Na	*O*-deacylated lipid A Positive ion mode
19.	1520.106	[M+2Na−H]^+^	1520.0191	Tetraacyl	2 × GlcN3N 1 × GalA 2 × 16:0(3-OH) 2 × 14:0(3-OH) 2 × Na	*O*-deacylated lipid A Positive ion mode
20.	1526.154	[M+Na]^+^	1526.0685	Tetraacyl	2 × GlcN3N 1 × GalA 1 × 16:0(3-OH) 1 × 17:0(3-OH) 1 × 14:0(3-OH) 1 × 15:0(3-OH) 1 × Na	*O*-deacylated lipid A Positive ion mode
21.	1534.128	[M+2Na−H]^+^	1534.0057	Tetraacyl	2 × GlcN3N 1 × GalA 2 × 16:0(3-OH) 1 × 14:0(3-OH) 1 × 15:0(3-OH) 2 × Na	*O*-deacylated lipid A Positive ion mode
22.	1548.129	[M+2Na−H]^+^	1548.0504	Tetraacyl	2 × GlcN3N 1 × GalA 1 × 16:0(3-OH) 1 × 17:0(3-OH) 1 × 14:0(3-OH) 1 × 15:0(3-OH) 2 × Na	*O*-deacylated lipid A Positive ion mode
23.	1578.089	[M+Na]^+^	1578.0035	Tetraacyl	2 × GlcN3N 1 × GalA 2 × 16:0(3-OH) 2 × 14:0(3-OH) 1 × Na 1 × P	*O*-deacylated lipid A Positive ion mode
24.	1600.085	[M+2Na−H]^+^	1599.9855	Tetraacyl	2 × GlcN3N 1 × GalA 2 × 16:0(3-OH) 2 × 14:0(3-OH) 2 × Na 1 × P	*O*-deacylated lipid A Positive ion mode
25.	1622.056	[M+3Na−2H]^+^	1621.9674	Tetraacyl	2 × GlcN3N 1 × GalA 2 × 16:0(3-OH) 2 × 14:0(3-OH) 3 × Na 1 × P	*O*-deacylated lipid A Positive ion mode
26.	1636.081	[M+3Na−2H]^+^	1635.9830	Tetraacyl	2 × GlcN3N 1 × GalA 2 × 16:0(3-OH) 1 × 14:0(3-OH) 1 × 15:0(3-OH) 3 × Na	*O*-deacylated lipid A Positive ion mode
22.	1650.095	[M+3Na−2H]^+^	1649.9987	Tetraacyl	2 × GlcN3N 1 × GalA 1 × 16:0(3-OH) 1 × 17:0(3-OH) 1 × 14:0(3-OH) 1 × 15:0(3-OH) 3 × Na	*O*-deacylated lipid A Positive ion mode
23.	1340.160	B_1_^+^	1340.1676	Tetraacyl	1 × GlcN3N 1 × 16:0(3-OH) 1 × 14:0(3-OH) 1 × 28:0(27-oxo) 1 × 19:0cyc	Intact lipid A Positive ion mode
24.	1420.136	B_1_^+^	1420.1340	Tetraacyl	1 × GlcN3N 1 × 16:0(3-OH) 1 × 14:0(3-OH) 1 × 28:0(27-oxo) 1 × 19:0cyc 1 × P	Intact lipid A Positive ion mode
25.	1141.935	B_1_^+^	1141.8729	Triacyl	1 × GlcN3N 1 × 16:0(3-OH) 1 × 14:0(3-OH) 1 × 28:0(27-oxo) 1 × P	Intact lipid A Positive ion mode
26.	919.782	B_1_^+^	919.7709	Triacyl	1 × GlcN3N 1 × 16:0(3-OH) 1 × 14:0(3-OH) 1 × 19:0cyc	Intact lipid A Positive ion mode
27.	901.762	B_1_^+^−H_2_O	901.7603	Triacyl	1 × GlcN3N 1 × 16:0(3-OH) 1 × 14:0(3-OH) 1 × 19:0cyc - H_2_O	Intact lipid A Positive ion mode
28.	981.734	B_1_^+^−H_2_O	981.7266	Triacyl	1 × GlcN3N 1 × 16:0(3-OH) 1 × 14:0(3-OH) 1 × 19:0cyc 1 × P - H_2_O	Intact lipid A Positive ion mode
29.	999.734	B_1_^+^	999.7372	Triacyl	1 × GlcN3N 1 × 16:0(3-OH) 1 × 14:0(3-OH) 1 × 19:0cyc 1 × P	Intact lipid A Positive ion mode
30.	641.509	B_1_^+^	641.5099	Diacyl	1 × GlcN3N 1 × 16:0(3-OH) 1 × 14:0(3-OH)	*O*-deacylated lipid A Positive ion mode
31.	721.475	B_1_^+^	721.4726	Diacyl	1 × GlcN3N 1 × 16:0(3-OH) 1 × 14:0(3-OH) 1 × P	*O*-deacylated lipid A Positive ion mode
32.	703.473	B_1_^+^−H_2_O	703.4656	Diacyl	1 × GlcN3N 1 × 16:0(3-OH) 1 × 14:0(3-OH) 1 × P - H_2_O	*O*-deacylated lipid A Positive ion mode
33.	685.469	B_1_^+^−2H_2_O	685.4551	Diacyl	1 × GlcN3N 1 × 16:0(3-OH) 1 × 14:0(3-OH) 1 × P - 2H_2_O	*O*-deacylated lipid A Positive ion mode

B_1_—nomenclature of ions according to Domon and Costello (1988)

Monoisotopic masses and *m/z* values were calculated using a Molecular Weight Calculator, a part of the Waters SYNAPT G2-S*i* HDMS instrument software

**Fig. 3:**
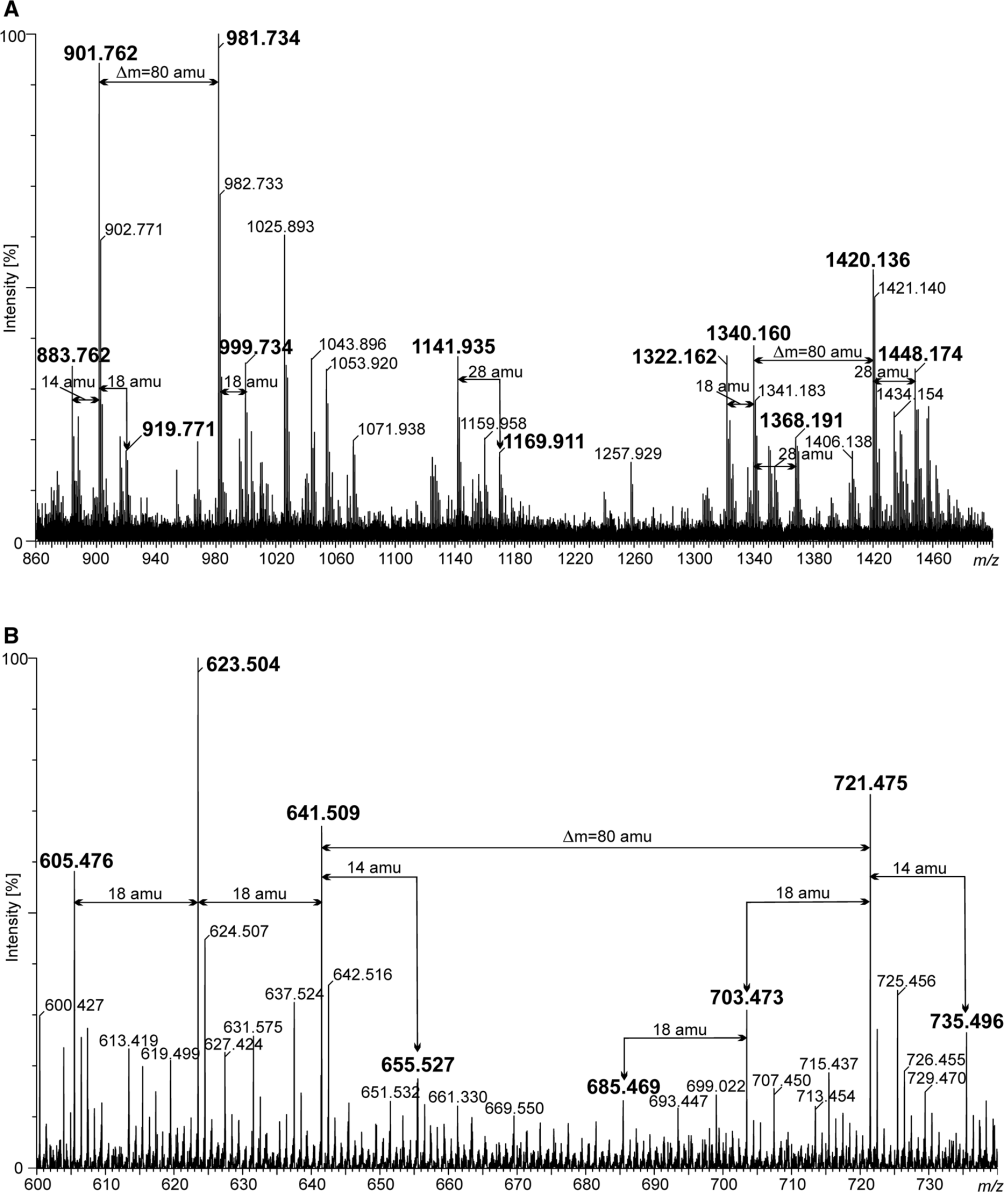
Positive ion mode MALDI TOF MS spectra. **a** Part of the spectrum containing B_1_^+^ ions derived from intact *P. trifolii* PETP02^T^ lipid A and **b** part of the spectrum containing B_1_^+^ ions derived from *O*-deacylated lipid A (with 1 M NaOH). Most of the selected ions in the spectra (marked in *bold*) are described in Table 2

**Fig. 4:**
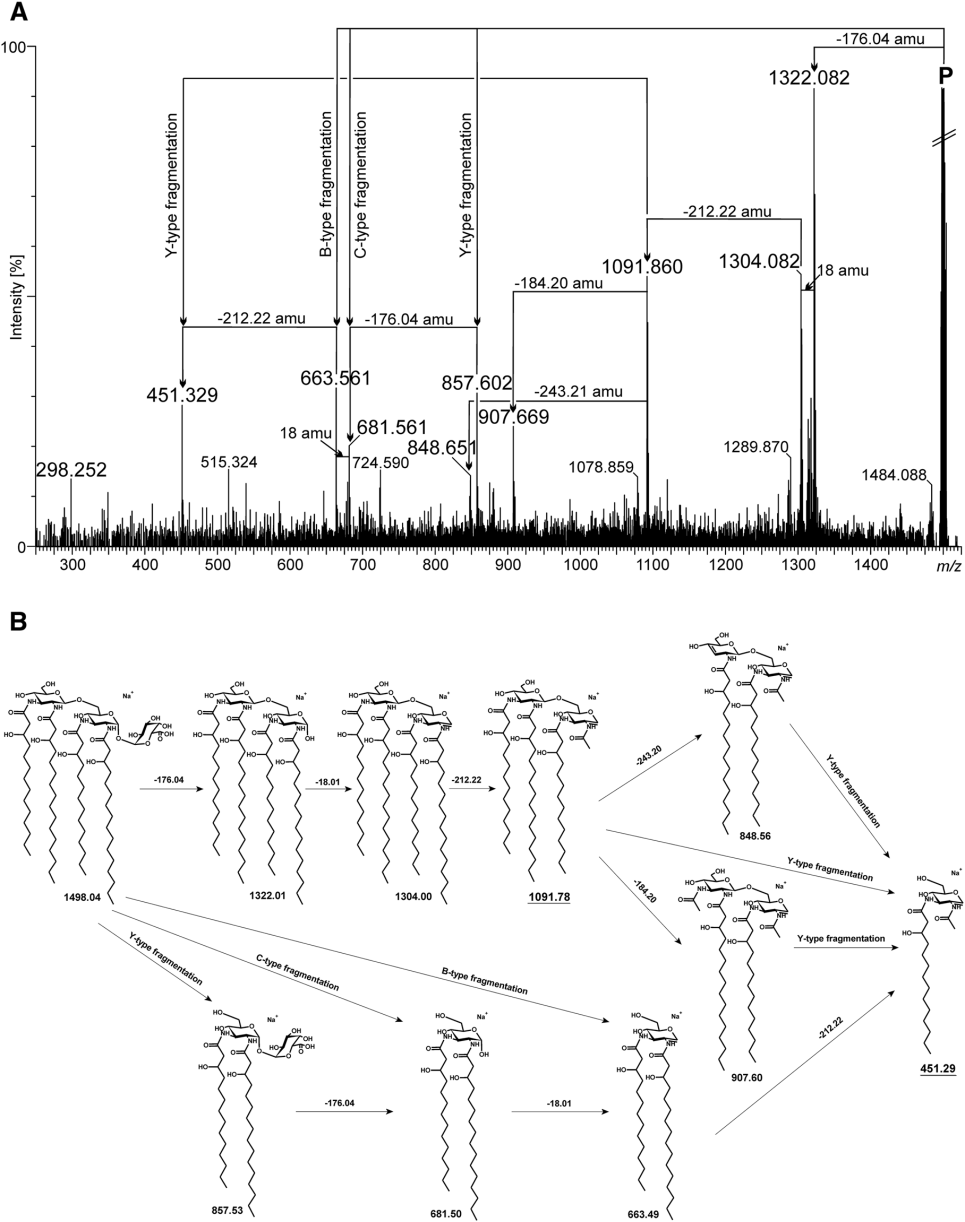
MALDI TOF MS/MS spectrum (**a**) of tetraacylated lipid A species at *m/z* 1498.12 (P—precursor ion) from *O*-deacylated *P. trifolii* PETP02^T^ lipid A. **b** Scheme of fragmentation of the precursor ion at *m/z* 1498.12. Indicative ions in determination of fatty acid distribution are *underlined*

Although MALDI is recognized as a soft ionization technique, it is known that, with the desorption of intact molecules/ions, in-source fragmentation occurs. Very informative B^+^ type lipid A ions can be found in the positive ion mode spectra of intact as well as *O*-deacylated lipid A preparations (Fig. 3a, b and Table 2). They were helpful in determination of the fatty acid distribution. The ions at *m/z* 1340.160 and 1420.136 unequivocally indicated that distal Glc*p*N3N was partly modified with phosphate (Δ mass = 80 amu). In addition, asymmetric distribution (4 + 2) of acyl residues on the disaccharide backbone of intact lipid A was deduced from the analysis of these ions. Thus, the ion at *m/z* 1340.160 can be interpreted as a tetra-acylated B_1_^+^ fragment bearing *N*-linked 16:0(3-OH) and 14:0(3-OH), and ester-linked lactobacillic and 27-oxooctacosanoic acids. The spectrum of *O*-deacylated lipid A included B_1_^+^ type ions as well. Ions at *m/z* 641.509 and 721.475 represented phosphorylated and unphosphorylated lipid A fragments composed of two fatty acids attached to Glc*p*N3N oxonium ions. Both these ions were accompanied by two mono- and doubly dehydrated product ions (Fig. 3b). Taking together the data from the analyses of intact and *O*-deacylated lipid A, it can be concluded that lipid A primary fatty acids were distributed symmetrically. MS/MS analyses were performed to assign the exact primary fatty acid positions at the reducing as well as non-reducing Glc*p*N3N. A set of ions was chosen from the *O*-deacylated *P. trifolii* lipid A MALDI TOF MS spectrum. The fragmentation pattern of the ion at *m/z* 1498.12 was selected to illustrate our results (Fig. 4a, b). This sodiated ion represents tetraacylated (exclusively with primary fatty acids) and devoid of phosphate species of *P. trifolii* lipid A (Table 2). After loss of Gal*p*A and water residues (176.04 and 18.01 amu, respectively), the precursor ion generated an ion at *m/z* 1304 containing a double bond between C-1 and C-2 of reducing Glc*p*N3N. The enamine to imine tautomerization of the compound resulted in elimination of aldehyde from the C-2 amide-bound fatty acid containing a free β-OH group (Choma et al. 2012; Silipo et al. 2014; Di Lorenzo et al. 2017). From two possibilities of Glc*p*N3N substitution with 3-OH fatty residues, only this one was represented and yielded a final ion at *m/z* 451.29 (Fig. 4a, b). The presence of this signal in the spectrum (and the absence of an ion peak at *m/z* 641.51) supported the placement of 16:0(3-OH) at C-2 and 14:0(3-OH) at C-3 of reducing as well as non-reducing Glc*p*N3N of the *P. trifolii* lipid A. The presence of other ions in the spectrum shown in Fig. 4b was explained by taking into consideration different fragmentation mechanisms as well (Fig. 4a).

According to Silipo et al. (2014), an ion corresponding to the pentaacylated lipid A, containing a VLCFA as the only secondary residue, is the best for determining the VLCFA position in the lipid A molecule. Intact lipid A from *P. trifolii* contained many hexaacylated species but there were few pentaacylated molecules (Fig. 1). Therefore, we used 25% ammonium hydroxide to remove partly and randomly *O*-linked fatty acids from the lipid A. Following the methodology proposed by Silipo et al. (2014), an ion at *m/z* 2000.34 with the composition (GlcN3N)_2_GalA_1_P_1_[C14:0(3-OH)]_2_[C16:0(3-OH)]_2_[28:0(27-OH)]_1_Na (P: represents a phosphate residue) was selected. Two B-type ions (B_2_-type at *m/z* 1806.4 and B_1_ at *m/z* 1165.9) were found (data not shown). Only the heavier one was accompanied by an ion originating from loss of ketene (∆ mass = 212.2 amu). These results allowed us to conclude that VLCFA (28:0(27-OH)) was linked to the hydroxyl of the primary fatty acid attached to position C-2′ of the lipid A backbone. These results are in agreement with the hitherto published VLCFA containing lipid A structures (Choma et al. 2017).

MALDI-TOF analysis clearly indicated that *P. trifolii* lipid A was very similar to its *M. huakuii* counterpart. Moreover, with respect to their backbones, both lipids A were identical. Thus, we decided to reduce the scope of the NMR analysis to ^1^H,^1^H homonuclear correlations sufficient to determine the anomeric configuration and the linkage positions within the lipid A backbone. The NMR data are listed in Table 3. The proton chemical shifts were assigned based on ^1^H,^1^H DQF-COSY, TOCSY, and NOESY spectra. Three proton signals were identified in the anomeric region of ^1^H NMR indicating that the *P. trifolii* lipid A backbone was built up of three sugar residues. Three spin systems: **A**, α-d-Gal*p*A; **B**, α-d-Glc*p*N3N; and **C**, β-d-Glc*p*N3N were described as well. The sequence of monosaccharides was established in the NOESY experiment by observation of the following interresidue correlations: **A**-1/**B**-1 (δ 5.19/5.04), **C**-1/**B**-6 (δ 4.39/3.76), and **C**-1/**B**-6′ (δ 4.39/3.84). The small values of ^*3*^ *J*_*H*-*1,H*-*2*_ coupling constants (<3 Hz) for Gal*p*A (**A**) and Glc*p*N3N (B) indicated an α-anomeric configuration of both residues. Thus, they were linked via an α-(1↔1)-glycosidic bond. Similar proton spectra were published for *M. huakuii* lipid A (Choma and Sowiński 2004).

**Table 3:** 500 MHz ^1^H NMR data of the sugar backbone of lipid A of *P. trifolii* PETP02^T^ (δ in ppm)

Sugar residue	*J*_1,2_ [Hz]	H-1	H-2	H-3	H-4	H-5	H-6	H-6′
α-d-GalA (**A**)	<3	5.19	3.91	4.03	4.28	4.20	–	–
α-d-GlcN3N (**B**)	<3	5.04	4.06	4.22	3.49	4.00	3.76	3.84
β-d-GlcN3N (**C**)	7.8	4.39	3.73	3.85	3.88	3.35	3.50	3.73

The ^31^P-NMR spectrum of the intact lipid A revealed a prominent signal with a chemical shift of 0.199 ppm observed in the solvent being a mixture of chloroform and methanol (proportion 2:1, v:v, respectively). These properties were indicative of the presence of phosphomonoester.

**Fig. 5:**
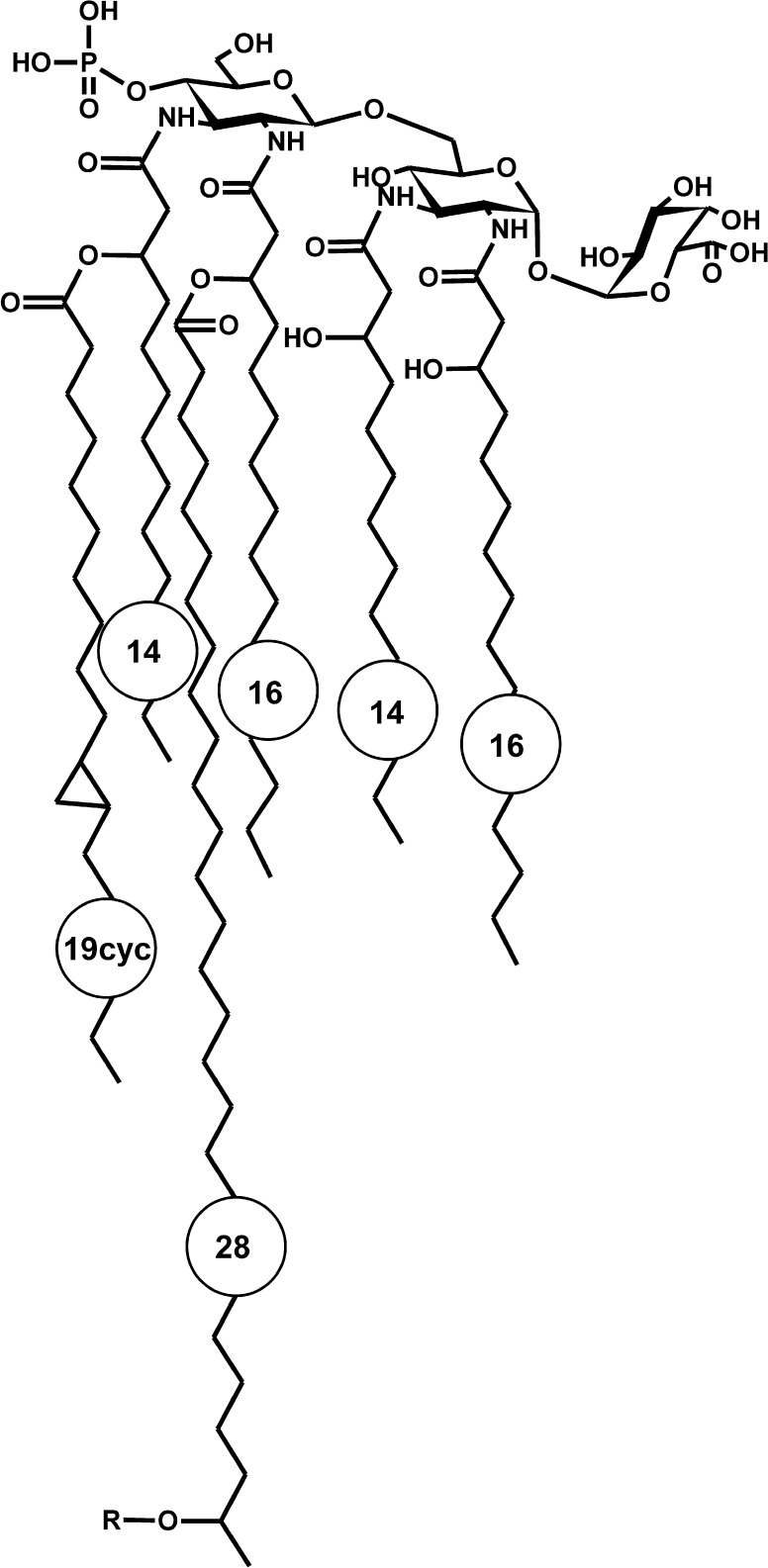
Proposed structure of the phosphorylated *P. trifolii* PETP02^T^ lipid A species decorated with: R - 3-methoxy-butyric residue, hydroxy-, or oxo- group, and containing most abundant 14:0(3-OH) and 16:0(3-OH) primary fatty acids

Based on all collected data, the following structure for lipid A being the lipophilic part of *P. trifolii* lipopolysaccharide was proposed (Fig. 5).

### Genetic background of lipid A biosynthesis pathway in *Phyllobacterium* species

In order to validate the experimental data related to the structure of *P. trifolii* PETP02^T^ lipid A obtained in MALDI-TOF mass spectrometry and NMR spectroscopy, in silico analyses of *Phyllobacterium* genomic sequences were performed for identification of putative genes encoding proteins engaged in lipid A biosynthesis. Although there is no available annotated genome sequence of *P. trifolii*, there are five high quality permanent draft genome sequences of strains belonging to the genus *Phyllobacterium*, among the genome sequencing projects registered in GOLD (Genomes OnLine Database) (Pagani et al. 2012). These strains comprise *Phyllobacterium* sp. OV277 (GOLD Project ID: Gp0136754), *Phyllobacterium* sp. UNC302MFCol5.2 (GOLD Project ID: Gp0039813), *Phyllobacterium* sp. YR620 (GOLD Project ID: Gp0136755), *Phyllobacterium* sp. YR531 (GOLD Project ID: Gp0012211), and *Phyllobacterium* sp. CL33Tsu (GOLD Project ID: Gp0115127). All five *Phyllobacterium* strains were isolated, cultured, and sequenced as part of three independent plant–microbe associated projects. Therefore, we used these genomic resources in searching for candidate genes of the *P. trifolii* lipid A biosynthetic pathway.

A comparative sequence analysis of the 16S rRNA gene indicated that *P. trifolii* is closely related to the members of the genus *Mesorhizobium* (Valverde et al. 2005). Our results have confirmed that the structure of *P. trifolii* lipid A is indeed similar (the backbone structure is even identical) to some mesorhizobial lipids A, i.e. *M. huakuii* IFO 15243^T^ and *M. loti* MAFF 303099 (Choma and Sowiński 2004; Brown et al. 2013). Thus, protein sequences of model rhizobial strain *M. loti* MAFF 303099 were used as queries in BLAST sequence similarity searching the draft genome sequences of the phyllobacteria, and then respective protein sequences were compared across their entire length with Needleman-Wunsch Global Align. Using this approach, we were able to recognize a set of putative genes coding for common enzymes required for the biosynthesis of lipid A (*lpxA*, *lpxC*, *lpxD*, *lpxH*, *lpxB*, *lpxK*, and *kdtA* (*waaA*)) as well as encoding specific enzymes involved in the structural modifications of lipid A found in some Gram-negative bacteria (*lpxE*, *rgtF*, and *acpXL*-*lpxXL*). Moreover, genes coding for enzymes converting Glc*p*N to Glc*p*N3N, necessary for biosynthesis of the lipid A disaccharide backbone and characteristic for bacteria from the genera *Mesorhizobium*, *Azorhizobium* and *Bradyrhizobium* (*gnnA* and *gnnB*), were identified (Table 4). Putative ORFs of the tested phyllobacterial species shared significant sequence similarity with the respective *M. loti* MAFF 303099 proteins (Table 4), strongly suggesting their common evolutionary ancestry—homology. We were not able to predict the putative homolog of α-(1↔1)-Gal*p*A transferase (*rgtF* gene) in the genome of *Phyllobacterium* sp. YR531. We found ORFs coding for putative 4′-phosphatase in the genomes of four *Phyllobacterium* strains (Ga0115491_102664, BR48DRAFT_1751, Ga0115492_1841, and Ga0073264_1478 in *Phyllobacterium* spp. OV277, UNC302MFCol5.2, YR620, and CL33Tsu, respectively), sharing 50–51% sequence similarity with the LpxF protein of *R. leguminosarum* bv. *viciae* 3841 (RL_RS08140), but we cannot identify homologs of RgtD, a putative 4′-GalA transferase of *R. leguminosarum* bv. *viciae* 3841 (RL_RS03600). Both LpxF and RgtD are involved in transfer of the Gal*p*A residue to lipid A during biosynthesis of LPS in *R. leguminosarum* bv. *viciae* 3841 (Brown et al. 2013).

**Table 4:** Sequence similarity of putative proteins required for the biosynthesis of *Phyllobacterial* lipid A

Predicted gene homolog	*gnnA*	*gnnB*	*lpxA*	*lpxC*	*lpxD*	*lpxH*
Putative function of encoded protein	catalyse the NAD-dependent oxidation of glucosamine 3-OH of UDP-GlcNAc	catalyse the subsequent transamination to form UDP 2-acetamido-3-amino-2,3-dideoxy–D-glucopyranose (UDP-GlcNAc3 N)	acyl-[acyl-carrier-protein]–UDP-N-acetylglucosamine O-acyltransferase	UDP-3-O-acyl N-acetylglucosamine deacetylase	UDP-3-O-(3-hydroxymyristoyl)-glucosamine N-acyltransferase	UDP-2,3-diacylglucosamine pyrophosphohydrolase
Strain						
*Mesorhizobium loti* MAFF303099 ORFs	MAFF_RS12135 (319)^a^	MAFF_RS30880 (380)	MAFF_RS03700 (279)	MAFF_RS07295 (316)	MAFF_RS03710 (351)	MAFF_RS04350 (260)
*Phyllobacterium* sp. OV277	Ga0115491_1011025 (354)^a^ 71% (78%)^b^	Ga0115491_1011023 (375) 74% (81%)	Ga0115491_11386 (277) 58% (73%)	Ga0115491_101745 (315) 63% (78%)	Ga0115491_11388 (352) 53% (71%)	Ga0115491_101496 (273) 67% (79%)
*Phyllobacterium* sp. UNC302MFCol5.2	BR48DRAFT_4614 (333) 75% (84%)	BR48DRAFT_4613 (373) 73% (81%)	BR48DRAFT_3598 (277) 57% (71%)	BR48DRAFT_2793 (313) 65% (80%)	BR48DRAFT_3596 (351) 53% (71%)	BR48DRAFT_2568 (273) 67% (78%)
*Phyllobacterium* sp. YR620	Ga0115492_0158 (333) 75% (84%)	Ga0115492_0159 (373) 74% (81%)	Ga0115492_2859 (277) 57% (71%)	Ga0115492_0672 (313) 65% (80%)	Ga0115492_2857 (351) 53% (71%)	Ga0115492_0900 (273) 67% (78%)
*Phyllobacterium* sp. YR531	PMI41_02102 (320) 79% (88%)	PMI41_02101 (374) 71% (80%)	PMI41_04833 (274) 55% (70%)	PMI41_01738 (316) 61% (76%)	PMI41_04835 (351) 51% (69%)	PMI41_01492 (273) 67% (78%)
*Phyllobacterium* sp. CL33Tsu	Ga0073264_3734 (333) 75% (84%)	Ga0073264_3733 (373) 73% (80%)	Ga0073264_2811 (277) 57% (71%)	Ga0073264_2497 (313) 65% (80%)	Ga0073264_2809 (351) 53% (71%)	Ga0073264_2268 (273) 67% (78%)

Predicted gene homolog	*lpxB*	*lpxK*	*lpxE*	*rgtF*	*kdtA*	*lpxXL*
Putative function of encoded protein	lipid-A-disaccharide synthase	lipid A 4′kinase	lipid A 1-phosphatase	α-(1,1)-GalA transferase	3-deoxy-D-manno-octulosonic-acid transferase (KDO transferase)	lipid A biosynthesis very long chain fatty acid acyltransferase to the β_2′_-position
Strain						
*Mesorhizobium loti* MAFF303099 ORFs	MAFF_RS03690 (390)	MAFF_RS33615 (341)	MAFF_RS01140 (271)	MAFF_RS01135 (549)	MAFF_RS33610 (438)	MAFF_RS05905 (328)
*Phyllobacterium* sp. OV277	Ga0115491_11384 (392) 53% (69%)	Ga0115491_106173 (343) 57% (71%)	Ga0115491_107147 (265) 35% (45%)	Ga0115491_101795 (561) 48% (62%)	Ga0115491_106172 (440) 71% (83%)	Ga0115491_101574 (310) 58% (72%)
*Phyllobacterium* sp. UNC302MFCol5.2	BR48DRAFT_3600 (391) 54% (70%)	BR48DRAFT_0877 (343) 57% (70%)	UNC302MFCol5.2 (261) 35% (46%)	BR48DRAFT_2841 (567) 48% (61%)	BR48DRAFT_0878 (440) 71% (82%)	BR48DRAFT_2640 (309) 58% (71%)
*Phyllobacterium* sp. YR620	Ga0115492_2861 (391) 54% (70%)	Ga0115492_2936 (343) 57% (69%)	Ga0115492_2582 (261) 36% (46%)	Ga0115492_0624 (567) 47% (61%)	Ga0115492_2935 (440) 71% (82%)	Ga0115492_0827 (309) 58% (71%)
*Phyllobacterium* sp. YR531	PMI41_04831 (389) 53% (69%)	PMI41_02675 (343) 55% (69%)	PMI41_04478 (254) 29% (44%)	–	PMI41_02676 (440) 70% (82%)	PMI41_01569 (311) 58% (72%)
*Phyllobacterium* sp. CL33Tsu	Ga0073264_2813 (391) 54% (70%)	Ga0073264_0583 (343) 57% (70%)	Ga0073264_0072 (261) 36% (46%)	Ga0073264_3479 (567) 48% (62%)	Ga0073264_0584 (440) 71% (82%)	Ga0073264_2342 (309) 58% (70%)

*Mesorhizobium loti* MAFF303099 protein sequences were used as queries in BLAST searches against protein sequences of various *Phyllobacterium* strains obtained from the IMG database

^a^ORF and number of encoded amino acids, refers to the entire Table

^b^% identity (% similarity), refers to the entire Table

The putative genes described display substantial conservation of organization (synteny and collinearity) among the tested phyllobacteria and in comparison to *M. loti* MAFF 303099 (Table 5). This was especially apparent with *lpxD*, *lpxA*, and *lpxB*, as well as *kdtA* and *lpxK* gene clusters, and the highly conserved *acpXL*-*lpxXL* region, typical for bacteria possessing lipid A molecules modified with VLCFAs (Choma et al. 2017). Similar to *M. loti*, the phyllobacterial *lpxC* and *lpxH* were not clustered with other genes related to LPS biosynthesis (Table 5).

**Table 5:** Comparison of the genetic organization of genes engaged in the biosynthesis of lipid A of *Mesorhizobium loti* MAFF303099 and *Phyllobacterium* sp. OV277

The *Phyllobacterium* sp. OV277 was arbitrarily chosen as a representative example of the genetic organization of respective regions, which was highly similar among the tested *Phyllobacterium* strains

There were noticeable differences found in the location of putative genes responsible for the transamination reaction of Glc*p*N (*gnnA* and *gnnB*), and structural modifications of Glc*p*N3N (*lpxE* and *rgtF*). In the case of phyllobacteria, orthologs of *gnnA* and *gnnB* are located close to each other but separately from the *lpxA*, *lpxB*, *lpxD* gene cluster, similar to *Bradyrhizobium japonicum* and *Brucella melitensis* (Sweet et al. 2004). In contrast, in *M. loti* MAFF 303099, the putative orthologs of these genes are separated both from each other and from the *lpxA*, *lpxB*, *lpxD* cluster. The *rgtF* and *lpxE* genes of *M. loti* MAFF303099 were clustered together with a putative *rgtE* gene (encoding putative bactoprenyl-phosphate Gal*p*A transferase) located upstream *rgtF*. Brown et al. (2013) suggested a functional relationship of these genes in the biosynthesis of lipid A α-(1↔1)-GalA. Interestingly, in all the tested phyllobacterial species, the putative *rgtE* and *rgtF* orthologs were neighboured, while the putative homolog of *lpxE* was distantly located from the above-mentioned ORFs.

## Discussion

In this study, we have described the structure of *P. trifolii* PETP02^T^ lipid A, which contains a trisaccharide carbohydrate backbone. This backbone comprises two d-Glc*p*N3N connected by a β-(1 → 6) glycosidic linkage and a d-Gal*p*A residue at position C-1. The substitution of the reducing end of lipid A by α-(1↔1)-d-Gal*p*A is unusual among bacteria and has been only described in lipids A from a few representatives of Gram-negative bacteria, including an associative diazotroph—*Azospirillum lipoferum* (Choma and Komaniecka 2008), and symbiotic bacteria—*M. huakuii* (Choma and Sowiński 2004), *M. loti* (Brown et al. 2013), a stalk-forming *Caulobacter crescentus* (Smit et al. 2008), and a thermophilic bacterium *Aquifex pyrophilus* (Plötz et al. 2000). The Glc*p*N3N disaccharide of *C. crescentus* and *A. pyrophilus* is bis-galacturonosylated with Gal*p*A located at positions C-1 and C-4′ (Plötz et al. 2000; Smit et al. 2008), whereas the *A. lipoferum* backbone does not possess the substituent at the reducing end of Glc*p*N (Choma and Komaniecka 2008). The *P. trifolii* lipid A trisaccharide backbone is partially phosphorylated at position C-4′ and has the same structure as that of *M. huakuii* IFO15243^T^ (Choma and Sowiński 2004). One can speculate that this may be related to the lack of an *rgtD* homolog in the genomes of the tested phyllobacterial species. The presence of phosphate, Glc*p*N3N, and Gal*p*A has also been reported in lipid A from *R. loti*, but with α-(1→4′)-Gal*p*A, not α-(1↔1)-Gal*p*A (Russa et al. 1995). The amino groups of both Glc*p*N3N of *P. trifolii* lipid A are symmetrically substituted by 3-hydroxy fatty acids, among which 14:0(3-OH) and 16:0(3-OH) dominate. Moreover, MS/MS data supported the placement of 16:0(3-OH) at C-2 and 14:0(3-OH) at C-3 positions of reducing as well as non-reducing Glc*p*N3N of the lipid A. Although the backbones of lipids A synthesized by phyllobacteria and mesorhizobia are identical, the substituting primary fatty acids are significantly different. This fact can be explained by the different basic metabolisms of both genera of bacteria. While the *Mesorhizobium* species synthesize and incorporate branched chain fatty acids into lipids (including lipids A), *Phyllobacterium* spp. produce predominantly straight chain fatty acids for cellular purposes (Tighe et al. 2000; Choma and Sowiński 2004; Valverde et al. 2005). As can be seen, the biosynthetic pathway of VLCFAs is not subject to this rule. *Mesorhizobium* as well as *Phyllobacterium* produces the same VLCFAs. Because of the distribution of ester-linked fatty acids (19:0cyc and 28:0-(27-OH/oxo)) restricted to the distal Glc*p*N3N, the entire lipid A is asymmetrically acylated, resembling *E. coli* and mesorhizobial lipids A (Raetz and Whitfield 2002; Choma and Sowiński 2004; Brown et al. 2013). This pattern of fatty acid distribution can be described by the formula 4 + 2. Hydroxyl groups at β positions of the primary fatty acids were additionally acylated by VLCFA and lactobacillic acid. The 27-hydroxyoctacosanoic acid could be partially acylated with 3-methoxybutyric acids. Lactobacillic acid is frequently found as a constituent of bacterial phospholipids. Especially phospholipids extracted from bacteria harvested in the stationary phase of growth are rich in this fatty acid as well as other cyclopropane fatty acids. To the best of our knowledge, cyclopropane fatty acids have not been found yet to be a component of LPS/lipid A. It can be assumed that, similar to phospholipids (Gronan and Cronan 1997), LPS containing unsaturated 18:1ω^7^ (vaccenic) acyl residue in the lipid A moiety undergoes modification in the bacterial outer membrane. This issue also remains to be resolved in future studies.

As mentioned above, we have found structural resemblance of lipid A of *P. trifolii* and *Mesorhizobium* and this observation seems to be reflected at the genomic level. Putative ORFs predicted for LPS biosynthesis in the phyllobacteria shared significant sequence similarity and overall similar gene organization with *M. loti,* with only minor differences. Further genetic analyses are required to answer the question how these differences affect the structure of *P. trifolii* lipopolysaccharide.
